# Emerging *Chryseobacterium indologenes* Infection in Indian Neonatal Intensive Care Units: A Case Report

**DOI:** 10.3390/antibiotics7040109

**Published:** 2018-12-14

**Authors:** Rishika Mehta, Ashish Pathak

**Affiliations:** 1 Department of Pediatrics, RD Gardi Medical College, Ujjain 456006, India; rishikamehta2390@gmail.com; 2 Department of Women and Children’s Health, International Maternal and Child Health Unit, Uppsala University, SE-751 85 Uppsala, Sweden; 3 Department of Public Health Sciences, Global Health—Health Systems and Policy, Karolinska Institutet, SE-171 76 Stockholm, Sweden; 4 International Centre for Health Research, Ujjain Charitable Trust Hospital and Research Centre, Ujjain 456006, India

**Keywords:** *Chryseobacterium indologenes*, blood stream infection, newborns, healthcare-associated pathogen

## Abstract

Antibiotic-resistant pathogens and nosocomial infections constitute common and serious problems for neonates admitted to neonatal intensive care units worldwide. *Chryseobacterium indologenes* is a non-lactose-fermenting, gram-negative, health care-associated pathogen (HCAP). It is ubiquitous and intrinsically resistant to several antibiotics. Despite its low virulence, *C. indologenes* has been widely reported to cause life-threatening infections. Patients on chronic immunosuppressant drugs, harboring invasive devices and indwelling catheters become the nidus for *C. indologenes*. Typically, *C. indologenes* causes major health care-associated infections such as pneumonia, empyema, pyelonephritis, cystitis, peritonitis, meningitis, and bacteremia in patients harboring central venous catheters. Management of *C. indologenes* infection in neonates is not adequately documented owing to underreporting, particularly in India. Because of its multidrug resistance and the scant availability of data from the literature, the effective empirical treatment of *C. indologenes* is challenging. We present an uncommon case of bacteremia caused by *C. indologenes* in a preterm newborn baby with moderate respiratory distress syndrome who was successfully treated. We also provide a review of infections in the neonatal age group. Henceforth, in neonates receiving treatments involving invasive equipment use and long-term antibiotic therapy, multidrug resistant *C. indologenes* should be considered an HCAP.

## 1. Introduction

Recently, *Chryseobacterium indologenes* has become a notorious multidrug resistant nosocomial pathogen [1]. In 1994, Vandamme and colleagues described a nonmotile, gram-negative, rod-shaped, aerobic, non-lactose-fermenting, catalase- and oxidase-positive bacillus, which was classified in the genus *Chryseobacterium* [2]. The family Flavobacteriaceae includes the genus *Chryseobacterium* [3]. Only six species of *Chryseobacterium* have been isolated from clinical specimens [3]. These species of *Chryseobacterium* are divided into two groups, IIa and IIb. *C. indologenes* is part of group IIb [3]. Among the six species, *Chryseobacterium meningiospecticum*, which is called *Elizabethkingia meningosepticum* according to the current taxonomical practices, is the most virulent. *C. indologenes* is minimally virulent [3]. Despite being ubiquitously present in soil, foodstuffs, and plants, *C. indologenes* is not part of the human microflora [3]. *C. indologenes* is typically isolated from hospital environments and cultured from specimens of sinks, indwelling vascular catheters, vials, feeding tubes, and other equipment in contact with fluids and water. Thus, C. *indologenes* can grow easily in liquid disinfectants [4]. *C. indologenes* is a rare human pathogen [4]. Most cases of *C. indologenes* have been isolated from immune-compromised individuals, patients with long-term indwelling devices, and patients on long-term antibiotics therapy [4]. *C. indologenes* sepsis can have uncommon presentations like pyonephritis, hepatobiliary infections, and meningitis [1]. Most cases of infections caused by *C. indologenes* in the pediatric population have been reported from children with congenital heart diseases or solid tumors [3], and least cases from neonatal age group have been reported. In this report, we describe a case of blood stream infection due to *C. indologenes* in a neonate with moderate respiratory distress syndrome and review previously reported infections caused by *C. indologenes* in the neonatal age group.

## 2. Case Presentation

A preterm male infant of a gestational age of 32 weeks and birth weight of 1550 g was delivered through a cesarean section owing to pregnancy-induced hypertension in the mother. He was transferred to the neonatal intensive care unit (NICU) because of labored breathing and cyanosis. No history of amniotic fluid leak or maternal fever was recorded. The heart rate and respiratory rate on admission to the NICU were 168/min and 72/min, respectively. Bilateral air entry decreased after auscultation, and a Silverman–Anderson distress score of 5, indicating moderate respiratory distress, was assigned to the infant [5]. A chest radiograph revealed bilateral white-out lungs with ground glass appearance. The nasal continuous positive airway pressure (CPAP) mode of ventilation was used, and his condition was managed using early rescue surfactant therapy with Neosurf (Cipla Ltd., Mumbai, India) at a dose of 5 mL/kg through the intubation–surfactant–extubation (InSuRE) technique [6]. The total leukocyte count was 7900/mm^3^ with 46% neutrophils and 51% lymphocytes. The neonate showed feed intolerance at 48 h of life and was kept nil orally for the next 24 h. A blood culture was sent for examination when the baby showed feed intolerance after 48 h of CPAP ventilation in view of suspected sepsis, as a part of sepsis screening. Further investigation revealed neonatal hyperbilirubinemia (total serum bilirubin, 13.4 mg/dL) on the third day of life, and the C-reactive protein level was 3.5 mg/L. 

The infant was provided with maintenance intravenous fluids, meropenem (40 mg/kg/day in two divided doses), and amikacin (15 mg/kg/day as infusion once a day) empirically, as per NICU policy. Respiratory secretions, oxygen requirements, and CPAP demands decreased after 16 h of life. Chest X-ray showed expansion of the lungs, and the infant was shifted to nasal prongs. 

The infant’s blood culture on nutrient agar showed the growth of smooth and circular 1–2 mm yellow-pigmented colonies after 24 h. When 10% KOH was decanted on the yellow colonies, they turned red because of the production of flexirubin [7]. Circular, low-convex, smooth, mucous colonies of 1–2 mm with beta-hemolysis developed on 5% sheep blood agar [3]. However, when cultured on MacConkey agar, no growth was observed. Biochemical reactions revealed that the microorganism was an oxidase-positive, catalase-positive, nonmotile, glucose non-fermenting, mannitol-negative, indole-positive, and urease-negative gram-negative bacillus. Using conventional biochemical reactions and the VITEK 2 system (bioMérieux India Pvt, Ltd., New Delhi, India), the organism was identified as *C. indologenes* [8]. Antimicrobial susceptibility testing was performed by both determining the minimal inhibitory concentration (MIC) value using the microdilution method and measuring the inhibition zone diameter on Mueller–Hinton agar medium aerobically at 35 ± 2 °C for 18–24 h by using Kirby–Bauer’s disk diffusion method. The MIC value was measured using the microdilution method. Clinical and Laboratory Standards Institute guidelines, 2018, for protocols for other non-enterobacteriaceae, non-fermenters other than *Pseudomonas aeruginosa* and members of the genera *Stenotrophomoas* and *Burkholderia* were used as reference for interpreting the results [9]. The isolate was resistant to piperacillin/tazobactam and colistin. The details of the MIC for various antimicrobial agents are shown in Table 1.

The antibiotic treatment was changed to Inj. cefoparazone with sulbactum (100 mg/kg in two divided doses) and Inj. ciprofloxacin (20 mg/kg in two divided doses) according to the culture report on day 5 of life and continued for 14 days. The condition of the infant improved, and he was discharged after completion of the antibiotic treatment.

## 3. Discussion

Globally, infections caused by *Chryseobacterium* spp. have been reported most frequently among elderly people (>65 years old) and least frequently among children <5 years old [1]. We summarized the published (reported) clinical cases in the neonatal age group in Table 2.

*C. indologenes* is resistant to chlorination and can survive in the water supplied from municipals sources [3]. Historically considered a minimally pathogenic microorganism, *Chryseobacterium* has been known to cause perilous infections mostly in hospitalized patients. Risk factors include underlying medical illness, extremes of age (newborn or elderly patients), immune-compromised condition, presence of indwelling intravascular devices, and prolonged exposure to broad-spectrum antibiotics [1,2,3,4,14,16]. *Chryseobacterium* grows on wet and humid surfaces in hospitals and in catheters containing fluids, such as central venous catheter, tracheostomy tubes, and feeding tubes [1,3,4,16]. Protease activity and production of biofilm by *C. indologenes* appear to be mechanisms relevant to its virulence; however, the exact mechanism of pathogenicity is not well documented [4]. Selecting an effective drug for the empirical treatment of the infections caused by *C. indologenes* is difficult owing to its narrow antibiotic spectrum susceptibility.

Production of β-lactamases makes this organism resistant to most β-lactam drugs, including the carbapenems and aztreonam [17]. *C. indologenes* shows maximum susceptibility to the newer quinolones like gatifloxacin, levofloxacin, and cotrimoxazole (95%).

It is moderately susceptible to piperacillin and tazobactam (90%) and less susceptible to ciprofloxacin, cefepime, ceftazidime, piperacillin, and rifampin (85%) according to surveillance data from Europe and the United States [1]. It is resistant to β-lactams, aminoglycosides, chloramphenicol, linezolid, and glycopeptides [17]. In our case, the isolate was resistant to colistin and piperacillin/tazobactam but was sensitive to other antibiotics, as shown in Table 1, including ciprofloxacin. Our patient responded well to ciprofloxacin. It is therefore recommended that clinicians should rely on the MIC values in the culture report to treat patients with *C. indologenes* infections, and microbiology laboratories should report the MIC values of ciprofloxacin along with those of newer quinolones like gatifloxacin and levofloxacin. In health care settings where colistine and tigecycline use is high for coverage of carbapenem-resistant pathogens, *C. indologenes* is emerging as a health care-associated pathogen (HCAP) [18]. However, fewer cases have been reported in neonates than in adults till date, which may be due to a relatively low frequency of comorbidities in the pediatric population [15]. 

## 4. Conclusions

In conclusion, this case shows that *C. indologenes* should be added to the list of HCAPs causing neonatal sepsis in neonates who receive long-term broad-spectrum antibiotics and in those who require invasive equipment use. The spread of this HCAP can be controlled by strict compliance with hand hygiene and by changing the equipment used for administering humidifying gases every 24 h [19].

## Figures and Tables

**Table 1:** Antimicrobial susceptibility of *Chryseobacterium indologenes* isolated from the patient’s blood culture.

S. No	Antibiotics	MIC (μ/mL)	Interpretation
1	Cefoperazone/ Sulbactum	<=8	Sensitive
2	Piperacillin/Tazobactam	>=128	Resistant
3	Imipenem	<=0.25	Sensitive
4	Meropenem	<=0.25	Sensitive
5	Amikacin	<=2	Sensitive
6	Gentamicin	<=1	Sensitive
7	Ciprofloxacin	0.5	Sensitive
8	Aztreonam	>16	Sensitive
9	Colistin	>=16	Resistant

MIC: minimal inhibitory concentration.

**Table 2:** Characteristics of neonatal cases caused by *C. indologenes.*

S.No.	Age/Sex	Underlying Condition	Medical Device	Infection Type	Treatment	Outcome	Reference
1	36 week newborn/NR	Prematurity	Ventilator	Bacteremia	Cefoperazone/Sulbactam	Survived	Sudharani et al. [10]
2	20 day/M	Complex congenital heart disease	Ventilator	VAP	Piperacillin/Tazobactam	Survived	Calderon et al. [11]
3	8 day/F	None	None	Meningitis	Cefepime	Survived	Hendaus et al. [12]
4	6 day/F	SGA	None	Meningitis, Sepsis, Bacteremia	Ciprofloxacin, TMP-SMX	Survived	Eshwara et al. [13]
5	10 day/F	Complex Congenital heart disease	Central Catheter	Bacteremia	Ciprofloxacin, Imipenem	Survived	Alford et al. [14]
6	18 day/M	Congenital diaphragmatic hernia	Oscillatory Ventilator	VAP	Levofloxacin, TMP-SMX	Survived	Smith et al. [15]
7	32 week newborn/M	Prematurity/RDS	CPAP	Bacteremia	Ciprofloxacin, Cefoperazone/Sulbactam	Survived	Present case

SGA: small for gestational age, CPAP: continuous positive airway pressure, VAP: ventilator-associated pneumonia, TMP–SMX: Trimethoprim–Sulfamethoxazole, RDS: Respiratory Distress Syndrome
